# Synaptic dysfunction in Down syndrome: an emerging role for circulating β2-microglobulin

**DOI:** 10.1038/s41392-023-01493-6

**Published:** 2023-05-29

**Authors:** Xu-Qiao Chen

**Affiliations:** Department of Neurosciences, University of California San Diego, La Jolla, CA 92093 USA

**Keywords:** Neurodevelopmental disorders, Pathogenesis

Recent research by Gao et al. published in *Cell* proposes that circulating β2-microglobulin impairs synaptic function through antagonizing NMDA receptors in two mouse models of Down syndrome (DS), providing a potentially novel therapeutic target to combat cognitive deficits in the early stage of DS.^1^

DS is caused by the presence of all or part of an extra human chromosome 21 (HSA21) and is associated with developmental and aging-associated changes. Intellectual disability is common in children with DS in the mild to moderate range.^2^ Although differences in brain development are correlated with cognitive deficits in children and young adults with DS, the changes driving cognitive dysfunction are unclear. Moreover, DS has a markedly increased risk for Alzheimer’s disease (AD). All adults with DS demonstrate AD-associated neuropathologies by age 40 and develop dementia mostly by age 60.^2^ The study by Gao et al. focused on cognitive dysfunction in the young adult stage of the two mouse models (Dp16 and Ts65Dn) of DS.

Parabiosis studies in these two mouse models revealed the impact of vascular systemic environment on synaptic dysfunction. Intravenous injection of plasma from the subjects with DS, but not non-DS siblings, into immunodeficient B-NDG mice induced similar synaptic deficits and deficient cognition-related behaviors, pointing to a negative effect of DS plasma on synaptic function.^1^ β2-microglobulin (B2M), a hub in the immune network in DS plasma, was identified with B2M levels significantly increased in both plasma and brains in both human and DS mouse models. Consistently, B2M-depleted DS plasma lost the capacity to compromise the synaptic function. Conversely, intravenous injection of B2M proteins in wild-type mice induced a parallel increase of B2M levels in both hippocampus and plasma and recapitulated the impacts of DS plasma on synaptic function.^1^

HSA21 harbors more than 200 protein-coding genes, including four genes encoding interferon receptors.^2^ In vitro MEF cultures conducted by Gao et al. suggest that active interferon signaling may play a role in driving the upregulation of B2M.^1^ However, direct evidence is needed to clarify the essential role of aberrant interferon signaling through the normalization of gene dose for interferon receptors in the context of DS. Additionally, the contributions of other HSA21 genetic factors cannot be excluded.

**Fig. 1:**
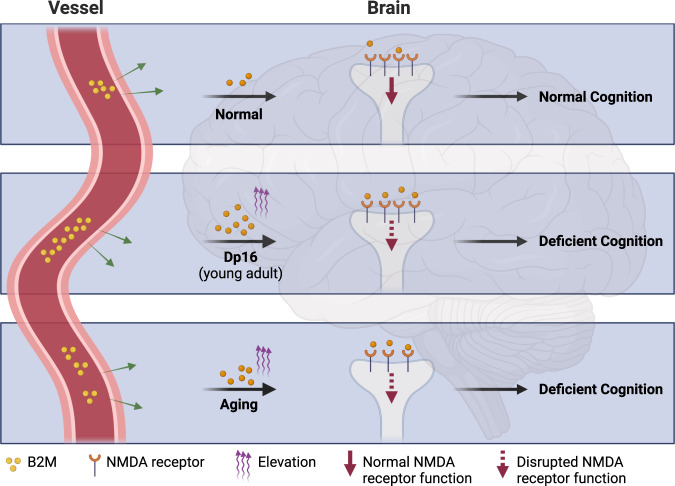
B2M links synaptic dysfunction in DS and aging. In both aging and DS conditions, B2M levels increase in both plasma and brain. During the young adult stage of the Dp16 mouse model of DS, the increased circulating B2M, possibly driven by hyperactive interferon signaling, specifically antagonizes the function of NMDA receptors, contributing to synaptic and cognitive dysfunction. In normal aging, previous studies have shown that increased circulating B2M impairs cognitive function by affecting deficient neurogenesis. Gao et al.^1^ have expanded on these findings by demonstrating that blocking the B2M-NMDA receptor interaction significantly restores synaptic function in aged wild-type mice. This suggests that the pro-aging effect of B2M is at least partly mediated by B2M-mediated antagonism of NMDA receptors. In addition, NMDA receptor subunits decrease with aging, further weakening NMDA receptor function. However, it remains unclear whether the mechanism of B2M-driven synaptic dysfunction proposed by Gao et al. is involved in the development of dementia in DS. Therefore, further research is needed to fully understand the underlying mechanisms and to develop effective therapeutic interventions. Created with BioRender.com

Both genetic ablation of one copy of the B2m gene and antibody-mediated B2M neutralization restored the synaptic and cognitive function in the Dp16 mice^1^ supporting the beneficial effects of B2M downregulation. Additionally, cognitive deficits in DS were linked to an imbalance of excitation/inhibition. Interestingly, Gao et al. discovered that *B2m* gene haploinsufficiency also reversed the excitation/inhibition imbalance in the Dp16 mice.^1^ The researchers then showed that B2M can efficiently cross the blood-brain barrier in both wild-type and Dp16 mice, supporting the idea that circulating B2M plays a role in impairing synaptic function (Fig. 1). Importantly, peripheral depletion of B2M reversed synaptic and cognitive deficits,^1^ not only demonstrating the role of circulating B2M in driving synaptic dysfunction but also raising a strategy to reverse synaptic deficits by reducing peripheral B2M levels in DS.

Mechanically, the authors found that B2M specifically antagonizes the function of NMDA receptors, not AMPA receptors, by reducing agonist potency. This effect was observed without changes to the postsynaptic and membrane distribution of NMDA receptors^1^ (Fig. 1). As a pro-aging factor, B2M impairs cognitive function through the canonical pathway involving TAP1-MHC1.^3^ However, Gao et al. conducted experiments using B2M-treated knockout mice and observed comparable effects of B2M on synaptic function, excluding the involvement of TAP1 and MHC1.^1^ These findings suggest that B2M may act through different mechanisms in different conditions (aging vs. DS) to confer its negative impact on synaptic function.

In line with the electrophysiology data, the researchers further demonstrated that B2M can bind to NMDA receptor subunits, particularly GluN1 and GluN2A, and identified an 18-mer segment in the S2 loop of GluN1 as the minimal binding unit for B2M. The authors then developed an 18-mer blocking peptide that effectively ameliorated the synaptic dysfunction induced by B2M treatments and in Dp16 brains. They also found that intrahippocampal injection of the blocking peptide significantly restored synaptic function in aged wild-type mice.^1^ These findings suggest that the systematic upregulation of B2M levels links aging and DS, given the pro-aging role of B2M^3^ and the accelerated aging observed in adults with DS (Fig. 1). Notably, NMDA receptor subunits are known to decline with aging, which has been associated with memory decline.^4^ Altered levels of ligands and dysregulated regulation of NMDA receptors are additional factors that contribute to impaired NMDA receptor function in both normal physiological processes and pathological conditions. However, Gao et al. pointed out that the pro-aging effect of B2M is at least partly mediated by the antagonism of NMDA receptors.

Deficits in neurogenesis, neuronal migration, maturation, and synaptogenesis during both prenatal and postnatal stages contribute to the intellectual dysfunction observed in children and young adults with DS. In the study by Gao et al., changes in plasma B2M levels in mouse models were found to parallel those observed in humans with DS. However, it remains to be determined whether the study’s findings can be extended to humans. Moreover, as DS mice exhibit both neuronal and synaptic deficits in early developmental stages, it is important to investigate how these mechanisms relate to the B2M-driven synaptic dysfunction in future studies. Cognitive function is stable over several decades in children and young adults with DS until the appearance of the common feature of AD-associated dementia. Therefore, further investigations are required to determine whether the mechanism of B2M-driven synaptic dysfunction proposed by Gao et al. is involved in the development of dementia in DS, particularly in the context of AD-related cerebral amyloid angiopathy which can disrupt the blood-brain barrier and increase the accessibility of circulating B2M to the brain. On the other hand, during the AD stage in DS, deficits in cognitive function are well correlated with AD-associated synaptic and neuronal loss.^5^ These mechanisms may synergize with other synaptic function deficits, including B2M-driven synaptic dysfunction, to contribute to the development of dementia in DS.

In conclusion, the study by Gao et al. has identified a potential therapeutic target for ameliorating cognitive dysfunction in individuals with DS by targeting circulating B2M or B2M-NMDA receptor interaction. This could have implications not only for the early stage of DS but also for the AD stages of DS as well as other neurodegenerative diseases. Further research is needed to fully understand the mechanisms involved and to develop effective therapeutic interventions.
